# Investigation of the Relationship between Spontaneous Abortion, Serum Pesticides, and Polychlorinated Biphenyl Levels

**DOI:** 10.3390/toxics11110884

**Published:** 2023-10-27

**Authors:** Jale Akgöl, Mine Kanat Pektaş

**Affiliations:** 1 Department of Medical Pharmacology, Faculty of Medicine, Afyonkarahisar Health Sciences University, Afyonkarahisar 03030, Turkey; 2 Department of Obstetrics and Gynecology, Faculty of Medicine, Afyonkarahisar Health Sciences University, Afyonkarahisar 03030, Turkey; mine.pektas@afsu.edu.tr

**Keywords:** pesticide, polychlorinated biphenyls, spontaneous abortion, reproductive toxicity, endocrin disrupters

## Abstract

Occupational and environmental chemical exposure have been associated with adverse reproductive consequences. This study investigates the relationship between spontaneous abortion and blood pesticide and polychlorinated biphenyl (PCB) levels. A survey was conducted, and blood samples were collected from 200 patients, consisting of 100 cases with spontaneous abortion and 100 cases with normal deliveries. A total of 150 different pesticides, including organophosphates, organochlorines, carbamates, and pyrethroids, were screened in the collected blood samples and analyzed quantitatively using Tandem mass spectrometry—specifically in combination with liquid chromatography and gas chromatography–tandem mass spectrometry methods. Eight types of PCBs were analyzed with the gas chromatography-tandem mass spectrometry method. The groups were compared based on these analyses. The mean age of the participants was 28.09 ± 4.94 years. In 59% of the spontaneous abortion group, 5.05 ± 1.97 chemicals were detected in different amounts. (*p* < 0.05). Analysis of the samples identified the presence of β-Hexachlorocyclohexane (β-HCH), delta-hexachlorocyclohexane (δ HCH), Hexachlorobenzene (HCB), Pentachlorobiphenyl-28 (PCB-28), Pentachlorobiphenyl-52 (PCB-52), o,p′-Dichlorodiphenyldichloroethylene (o,p′-DDE), p,p′-Dichlorodiphenyldichloroethylene (p,p’DDE), o,p′-Dichlorodiphenyldichloroethane (o,p′-DDD), p,p′-Dichlorodiphenyldichloroethane (p,p′-DDD), Pentachlorobiphenyl-118 (PCB-118), Pentachlorobiphenyl-101 (PCB-101), Pentachlorobiphenyl-153 (PCB-153), Pentachlorobiphenyl-138 (PCB-138), Pentachlorobiphenyl-202 (PCB-202), Pentachlorobiphenyl-180 (PCB-180) as well as Fibronil, Buprimate, Acetoclor, Acemiprid, Pentimanthalin, and Triflokystrobin. The spontaneous abortion group had significantly higher exposure to PCB-101, PCB-52, PCB-138, and δ-HCH (*p* < 0.05). Women included in the study had high pesticide and PCB exposure rates. Many of the blood samples contained multiple pesticides with endocrine-disrupting effects. Higher exposure to organochlorine compounds in the serum was identified in the group with spontaneous abortions.

## 1. Introduction

Spontaneous abortion (SAB), or miscarriage, is defined as losing a pregnancy before 20 weeks without external intervention. The literature reports that the incidence rate of SAB is between 12.8% and 13.5% of clinically recognized pregnancies, and this incidence has shown an upward trend over the years [1]. SAB is a pregnancy complication with a multifactorial etiology involving various factors [2]. Chromosomal anomalies account for 50% of the causes of SAB. Environmental factors such as maternal age; anatomical, endocrinological, and immunological disorders; infections; smoking; alcohol consumption; placental anomalies; heavy metals; radiation; and pesticides can also contribute to SAB [3,4,5].

Pesticides constitute a diverse group of heterogeneous chemicals that enhance food production efficiency and reduce foodborne and vector-borne diseases to meet growing food demand. Pesticides are considered indispensable to agriculture, and the absence of pesticide use in fruit production today is associated with yield losses of up to 80% [6]. Pesticides used to combat weeds and crop pests not only affect organisms like birds, fish, beneficial insects, and non-target plants but also disperse into the ecosystem through air, water, and soil, showing adverse effects on human health across various systems, including hematological, endocrine, and neurological systems [7].

Although the lack of definitive biological markers makes it difficult to establish a cause-and-effect relationship, numerous studies investigate environmental pollutants’ potential effects on SAB among women of reproductive age [8,9,10]. Endocrine disruptor chemicals are more frequently linked to problems with female reproduction, including irregular menstruation, early menopause, delayed menarche, ovarian dysfunction, reduced fertility, and unfavorable pregnancy outcomes. Exposure to agricultural and industrial chemical toxic agents during the early stages of pregnancy can lead to various effects, such as intrauterine growth restriction, fetal and postnatal death, congenital disabilities, preterm birth, cognitive development issues, and immunological dysfunction [11,12,13]. Studies, particularly those involving women working in agricultural fields, support the hypothesis of a relationship between pesticides and SABs [14,15,16]. 

Persistent organic pollutants (POPs) are carbon-based organic chemical compounds. Polychlorinated biphenyls (PCBs) are persistent organic pollutants with industrial uses and are known to have highly toxic, endocrine-disrupting effects. PCBs have been linked to many reproductive problems [17,18].

Evaluating the role of widespread and high-level occupational and environmental chemical exposure in the etiology of SAB is critical. This study investigates the quantitative and qualitative relationship between several pesticides, PCBs, and SAB regarding reproductive toxicity.

## 2. Materials and Methods

### 2.1. Study Design 

This study is a prospective case–control study. The sample size for the study population to be included in the study scope was aimed to be 98 patients and 98 control subjects, with a research effect size of 0.52, an alpha error rate of 0.05, and a power of 95%, assuming a 1:1 ratio for the compared groups. The study’s recruitment was concluded when the study group reached 100 patients and 100 controls [19]. Participants’ blood samples were obtained from women who presented to the Obstetrics and Gynecology Clinic at the Afyonkarahisar Health Sciences University Faculty of Medicine. Blood samples were collected between September 2020 and December 2022. The study started after the decision of the Afyonkarahisar University Clinical Researches Local Ethics Committee on 8 January 2021 and was numbered 2021/81.

### 2.2. Inclusion and Exclusion Criteria for Study Groups

The inclusion criteria for the case group in this study were as follows: presenting to Afyonkarahisar Health Sciences University Faculty of Medicine due to SAB, aged between 18 and 35, not having a history of consanguineous marriage, non-smoker and non-alcohol user, absence of chronic diseases, no history of medication use during the first three months of pregnancy, and no history of cervical insufficiency. The inclusion criteria for the control group, consisting of women who had healthy deliveries, were as follows: successful completion of pregnancy within the expected timeframe, aged between 18 and 35, absence of chronic diseases, non-smoker and non-alcohol user, no history of consanguineous marriage, and no treatment procedure related to pregnancy.

### 2.3. Study Protocol

Study participants were administered a questionnaire to inquire about their residential proximity to agricultural areas, age, education, occupational background, and pregnancy history, including number of births, delivery methods, gestational weeks, dietary habits, and exposure to agricultural chemicals. This survey included some additional questions. In the nutritional habits inquiries, we questioned the consumption amounts of meat, fish, eggs, milk, vegetables, and fruits on a monthly, weekly, and daily basis. Additionally, we asked how they clean raw vegetables and fruits. In order to measure their level of knowledge about pesticides, we asked five questions about their awareness of the effects and harms of pesticides on general health. We made a knowledge score by giving 1 point for each correct answer. 

Two 3 mL blood samples (K2EDTA 5.4 mg, B.D. Vacutainer, Plymouth, UK) were promptly collected from both groups and stored at −20 °C. Pesticide and PCB analyses of biological samples were performed in the Çukurova University Hospital Forensic Toxicology Laboratory. Pesticides screened in this study consisted of insecticides, fungicides, and herbicides used for grains, wheat, barley, potatoes, poppy, cucumbers, cherries, and apples produced throughout the province, as well as pesticides widely used in Turkey. A total of 150 different pesticides and eight types of PCBs were screened in serum. Pesticides banned for use due to potential inappropriate application and contact were also included in the screening. Organophosphate pesticides were analyzed using Tandem mass spectrometry—especially in combination with liquid chromatography (LC/MS-MS), while organochlorine pesticides and PCBs were analyzed using gas chromatography–tandem mass spectrometry (GC/MS-MS) with appropriate protocols (Table S1 and Table S2).

### 2.4. Statistical Analysis

The obtained data were analyzed using the IBM SPSS 25.0 software package. The normal distribution suitability of numerical variables was investigated using the Shapiro–Wilk Test. For normally distributed numerical variables, comparisons between groups were conducted using either the *t*-Test or One-Way Analysis of Variance (ANOVA), while for non-normally distributed variables, group comparisons were made using the Mann–Whitney U Test or the Kruskal–Wallis H Test. The relationships between categorical variables were examined using the Chi-Square Test, and the correlation between continuous variables was explored using Pearson Correlation Analysis. Descriptive statistics were used for data evaluation, while estimated relative risk was used for univariate analyses. Statistical differences were considered significant at a *p* value of less than 0.05.

## 3. Results

A total of 200 samples were included in the chromatographic analysis. The mean age of the participants was 27.86 ± 4.5 years (min–max: 18–35 years). Among the participants, 38.5% completed primary school, and 53.5% completed middle or high school. In total, 9% of participants resided within agricultural areas, 30% resided near agricultural lands, and 59.5% lived in urban or suburban areas away from agricultural lands (Table 1).

Exposure to only organophosphate (OF)-derived pesticides was identified in 3%, exposure to only organochlorine (OC) pesticides and PCBs was identified in 50.5%, and exposure to both OC and OF pesticides and PCBs was identified in 5.5% of participants. Residues of one or more pesticides and PCBs were detected in 59% of the participants. When chemicals were examined independently, it was discovered that the SAB group had significantly higher exposure to PCB-101, PCB-52, PCA-138, and δ-HCH (*p* < 0.05). The odds ratio in the 95% confidence interval for these chemicals is, respectively, 16.11 [CI: 2.076–125], *p* = 0.000; 2.524 [CI: 1.04–6.113], *p* = 0.036; 1.991 [CI: 0.981–4.041], *p* = 0.05; and 2.603 [CI: 1.257–5.391]; *p* = 0.009, respectively.

The incidence of SAB was significantly higher among women living within or near agricultural lands (62.8%) compared to women living farther away from agricultural lands (42%) (*p* < 0.05). The mean Body Mass Index (BMI) of the participants was 26.8 ± 2.8. Among the control group, 59% experienced normal childbirth. Of the SAB cases within the first 12 weeks of pregnancy, 73% occurred, while the rest occurred between 12 and 20 weeks. In the control group, 50% of infants were born weighing between 3000 and 3500 g. Among the participants, 10.5% were in their first pregnancy, and 32.5% were in their second pregnancy when seeking medical attention. Among women who experienced SAB, 31% had a history of pesticide exposure, while this rate was 8% among women who had normal births, and this difference was found to be statistically significant (*p* < 0.05) (Table 2).

The total number of pregnancies was significantly higher in the SAB group compared to the control group (*p* < 0.05); furthermore, 47% of SAB cases and 43% of normal birth cases had a history of at least one miscarriage (*p* > 0.05). 6.5% of all participants had a history of recurrent abortion, and 7.5% had a history of stillbirth, and this was significantly higher in the spontaneous abortion groups (*p* = 0.016; *p* = 0.000, respectively). There was no significant relationship between pesticide exposure and a history of miscarriage, recurrent abortion, or stillbirth (*p* = 0.537, *p* = 0.935, and *p* = 0.847, respectively). The distribution and comparison of OCP and PCB levels (ng/mL) in the case and control groups are presented in Table 3.

A set of three questions regarding agricultural products was used to assess the participant’s knowledge about the characteristics, protective measures, and disposal of agricultural products, with a total score of 15 points. There were no significant differences in the answers between the groups (*p* > 0.05) (min–max: 8–15) (Q1–Q3/9–15).

Participants were asked about their daily, weekly, and monthly food consumption habits. No significant relationship was found between food consumption habits and pesticide exposure (*p* > 0.05). 

It was determined that 29.7% of women who had detectable levels of pesticides in their blood washed raw fruits and vegetables prior to consumption by soaking them in vinegar water, although this was not statistically significant. The rate of participants who washed fruits and vegetables by only rinsing with running water, soaking in hot water, soaking in vinegar water, or soaking in baking soda and water was significantly more prevalent in the normal birth group (*p* < 0.05). Additionally, 34% of the normal birth group washed fruits and vegetables with running water, and 29% of the SAB group consumed them by soaking them in vinegar water (*p* > 0.05). The normal birth group consumed more meat and fish than the SAB group (*p* < 0.05). No significant relationship was found regarding the consumption of fresh fruits and vegetables (*p* > 0.05). It was determined that 19.5% of participants have a history of exposure to agricultural products.

Despite being banned pesticides, Acetochlor, Thiometan, Acetamiprid, Pentimanthalin, Trifloxystrobin, DDT, and HCH were identified in the blood samples in this study.

**Figure 1:**
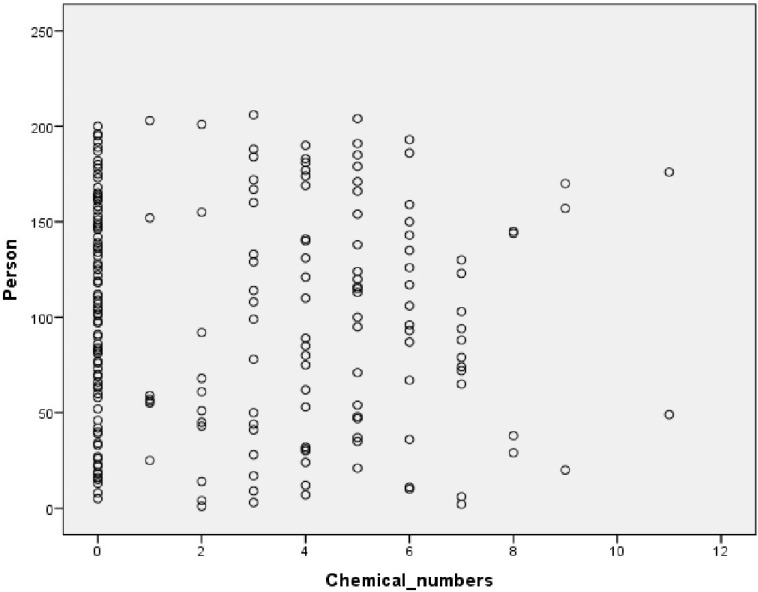
Scatter plot of residual chemical numbers (row) by person (column).

Among individuals who sought medical attention due to SAB, various levels of pesticides and PCBs were found in 59% of cases. SAB cases with detected levels of pesticides demonstrated the presence of at least 1 and up to 11 different types of chemicals, with an average exposure of 5.05 chemicals and a median value of 5 (Figure 1), with multiple types of pesticides simultaneously detected in the blood (Q1–Q3/4–6). In the control group, the mean exposure to 4.02 chemicals was detected with a median value of 4 (Q1–Q3 = 3–5). (Table 4).

Although there was slightly higher organochlorine compound exposure in the SAB group compared to the control group, there was no significant quantitative relationship between exposures.

Organochlorine compounds such as Beta HCH, δ HCH, HCB, PCP-28, PCP-52, o,p-DDE, p,p-DDE, o,p′-DDD, p,p′-DDD, PCB-118, PCB-153, PCB-138, PCB-202, and PCB-180 were detected in the blood samples of the participants. Organofluorine pesticides like Fipronil, Buprimate, Acetochlor, Acetamiprid, Pentimanthalin, and Trifloxystrobin were also detected. PCB-138, PCB-101, Gamma HCH, and PCB-52 were significantly higher in the SAB group compared to the control group (*p* < 0.05). Pendimethalin was found to be elevated in the group with normal births (*p* < 0.05).

## 4. Discussion

The pregnancy period, especially the first trimester, encompasses a critical phase of embryonic growth and development, making it a highly significant period. The relationship between toxic substances encountered during this period and reproductive health has been substantiated [20]. Endocrine-disrupting pesticides can exert their effects through many different mechanisms. Direct toxic exposure to reproductive tissues and hormones is one of these mechanisms. In addition, organophosphates, like carbamates and pyrethroids, may cause abnormalities in the female reproductive system by disrupting the hormonal axis due to anticholinesterase activity. As with organochlorine pesticides, endocrine-disrupting effects may occur through excessive stimulation of hormone receptors using pesticides with hormone-like activities. These abnormalities include decreased fertility, congenital disabilities, menstrual disorders, fetal growth restriction, or increased spontaneous abortions [21,22]. There are also studies linking pesticides to pregnancy losses by increasing placental oxidative stress [23]. Some publications in the literature and toxicology sources indicate that a woman encounters at least 60 toxic agents during pregnancy. However, assessing their potential effects is complex due to the scarcity of exposure data, the lack of suitable diagnostic markers, and the uncertainties associated with translating effects from animals to humans [9,24]. Research indicates an increase in reproductive health issues over the years. More than half of the participants in this study were found to have been exposed to at least one type of pesticide. While there was a higher number of pesticide exposures in the SAB group and a lower number in the control group, the lack of a significant difference between the groups can be attributed to the complexity of pesticide effects, which are influenced by factors such as the active substances, duration and quantity of exposure, gestational age, and the stage of gestational development when exposure occurs.

It has been observed that women who mention a history of past exposure tend to cluster in the SAB group. In the literature, various factors such as genetics, immunity, hormones, age, anatomical reasons, semen quality, and environmental factors are implicated in the etiology of SAB. The impact of pesticides on SAB has been particularly demonstrated in studies involving agricultural workers [25,26]. The Ontario Farm Family Health Study in Canada investigated 2110 women who were agricultural workers, 3936 pregnancies, and 395 SAB cases, revealing an association between early and late exposure and SAB [27]. A study of grape workers in India demonstrated a higher incidence of SAB associated with pesticide exposure [28]. Piazza et al. also demonstrated the reproductive toxicities of atrazine, polychlorinated and polybrominated biphenyls, dichlorodiphenyltrichloroethane (DDT), dichlorodiphenyldichloroethylene (DDE), and dichlorodiphenyldichloroethane (DDD) [29]. In this study, a variety of pesticides, including those banned for use, such as DDT, DDE, hexachlorocyclohexane (HCH), and hexachlorobenzene (HCB), were detected in both the SAB and normal birth groups. Due to the atmospheric distribution and dispersal properties of pesticides, it is understood that the issue is not solely limited to individuals working in agricultural fields. A 2011 study conducted in the Afyon province of Turkey investigated breast milk and detected various pesticides in the samples of 96% of the 80 participating women [30].

The collected blood samples revealed the presence of residues of organochlorine compounds, including Beta HCH, δ HCH, HCB, PCP-28, PCP 52, o,p-DDE, p,p′-DDE, o,p′-DDD, p,p′-DDD, PCB-118, PCB-153, o,p DDT, p,p DDT, PCB-138, PCB-202, and PCB-180, as well as OF pesticides such as Fipronil, Buprimate, Acetochlor, Acetamiprid, Pentimanthalin, and Trifloxystrobin. Organochlorine pesticides and PCBs are more prominently detected, especially due to their cumulative properties. Various studies have associated this group of pesticides, particularly DDT and DDE, with SAB etiology. Detecting elevated levels in the serum samples of women experiencing recurrent pregnancy losses is a significant factor in explaining the etiology of SAB [31,32].

In the scope of the study, PCB-138, PCB-101, Gamma HCH, and PCB-52 were significantly higher in the SAB group compared to the control group. Only Pentimanthalin was found to be elevated in the blood samples of six women who had normal births.

Polychlorinated biphenyls (PCBs) are organic pollutants that are widespread in the environment and vary in structure based on the number of attached chlorine atoms (e.g., PCB-28, -138, -153, -156, -170, and -180). PCBs have been used in various industries, including as pesticide additives. They have been associated with metabolic disorders, neurotoxicity, liver toxicity, and reproductive and developmental toxicity [33]. This study detected PCB-28, -52, -101, -118, -153, -138, -180, and -202 levels. PCB-52, -101, and -138 positivity were significantly higher in the SAB group.

PCBs and pesticide metabolites such as 1,1-dichloro-2,2-bis(p-chlorophenyl)ethylene (DDE) and 1,1-trichloro-2,2-bis(p-chlorophenyl)ethane (DDT) are hormonally active substances. High-chlorinated PCBs accelerate estrogen metabolism by inducing CYP enzymes, leading to anti-estrogenic effects. Due to their chemical structure resembling estradiol, low-chlorinated PCBs stimulate estrogen receptors and increase their activity. Hence, these compounds lead to reproductive toxicity through estrogenic or anti-estrogenic effects [34]. These effects suggest that these compounds could potentially affect fertility by causing risks such as disruption of the hormonal cycle, changes in menstrual function, or the formation of uterine masses, leading to decreased fertility and an increased likelihood of spontaneous miscarriages. In studies involving 135 women undergoing in vitro fertilization (IVF) treatment, it was found that PCBs, specifically PCB-118, had a negative impact on blastocyst activity [35].

Pendimethalin (3,4-Dimethyl-2,6-dinitro-N-(pentan-3-yl)aniline) is an effective herbicide used on weeds in crops like potatoes, cabbage, peas, and grains. It demonstrates its toxic effects by generating superoxide radicals that deplete the antioxidant system [36]. Genotoxic effects have been observed in analyses involving zebrafish [37]. In this study, Pendimethalin was found to be elevated in the group with normal births. Its cytotoxic and genotoxic actions at the cellular level are well understood, but additional research is required to determine its effects on tissues and organs [38,39].

Gamma HCH, also known as Lindane, is one of the isomers of HCH (hexachlorocyclohexane). The other two isomers are alpha and beta, and their production is no longer carried out. Gamma HCH is used as an insecticide in agriculture and also in the medical treatment of scabies. It belongs to the class of organochlorine pesticides and, when released into wastewater without proper filtration, can contaminate groundwater and soil, posing a persistent environmental pollutant harmful to both ecology and human health [40]. Since the Stockholm Convention, it has been banned in 52 countries and has restricted use in 33 countries. Prenatal exposure to Lindane has been studied in rats, demonstrating that Lindane exposure before birth leads to a persistent increase in cerebral cytochrome P450 (CYP) expression in young rats. This finding suggests that alterations in xenobiotic responses may contribute to the etiology of toxicity. Another study on nematodes has found toxicity associated with increased superoxide radicals and decreased antioxidant activity. Increased oxidative stress, elevated lipid peroxidation, and single-stranded DNA breaks were observed in fetal and placental tissues [41,42]. Numerous studies have reported a significant relationship between elevated serum Lindane levels and spontaneous miscarriages in women [43,44]. The significant elevation of Lindane levels in the SAB group in the present study is consistent with the literature.

According to the research data, 45% of women had a history of SAB. In women of reproductive age, 25–50% experience one or more miscarriages, and 10–15% of pregnancies end in miscarriage. These data are consistent with the literature. It is known that the majority of first-trimester miscarriages occur due to chromosomal anomalies. Human studies have also demonstrated the relationship between certain environmental pollutant-like pesticides, such as the phenoxy herbicide group pesticides, and chromosomal anomalies [45,46].

In this study, no differences were observed regarding age, educational status, pregnancy count, history of miscarriages, dietary habits, and pre-consumption washing and cleaning habits of raw vegetables and fruits. The rate of applying any cleaning treatment before consuming raw fruits and vegetables was significantly higher in the women who had normal births [47]. Residues in agricultural products should be below permissible limits. Various methods, like washing with ozone, acetic acid, citric acid, and carbonic acid, are used to remove residues. According to a study investigating the reduction in primiphos-methyl residues in bell peppers through different washing methods, it was observed that washing with acetic acid (vinegar) removes more residues compared to tap water [48]. Research shows that the most effective method for removing pesticides is washing with plenty of running water [49].

The main limitations of this study were the lack of specific timing information regarding the exposure period to pesticides, the uncertainty of whether the exposure was acute or chronic, and the absence of male factors. The inclusion criteria were tightened to reduce the impact of other factors on the link between SAB and pesticides.

## 5. Conclusions

Patients included in the study had high pesticide exposure rates. Many of the blood samples were found to contain multiple pesticides and PCBs with endocrine-disrupting effects. Higher exposure to organochlorine pesticides in the blood was identified in the group with spontaneous abortions. The detection of banned pesticides, including DDT, in the blood was noteworthy. It is known that pesticides not only lead to reproductive toxicity but also possess mutagenic, carcinogenic, and teratogenic effects. Inspections should be conducted regarding the detected banned substances, and individuals engaged in agriculture should be informed about conscious pesticide usage. More specific studies are needed to establish the relationship between the quantitative differences of pesticides, PCBs, and spontaneous abortion. Measurements of the level of endocrine disturbers may one day be helpful as a biomarker, thanks to studies on the effects and prognostic significance of pesticides or PCBs on spontaneous abortion.

## Figures and Tables

**Table 1:** Demographic characteristics of case (spontaneous abortion) and control groups.

Variables	The Group with Spontaneous Abortions (n = 100)	The Group with Normal Deliveries (n = 100)	*p* Value
	Mean ± SD	Mean ± SD	
Age	27.73 ± 4.5	27.99 ± 4.5	0.5
Weight	68.6 ± 8.0	70.5 ± 9.2	0.2
BMI (kg/m^2^)	25.9 ± 2.3	26.8 ± 3.2	0.021
Total Number of Pregnancies	3.01 ± 1.09	2.67 ± 0.5	0.027
Knowledge Level	12.3 ± 2.3	11.94 ± 2.6	0.3
Total Number of Chemicals Identified	5.05 ± 1.97	4.1 ± 2.06	0.007

*p* value = Mann–Whitney U Test; SD: Standard Deviation, BMI: Body Mass Index.

**Table 2:** Descriptive characteristics of the groups.

Descriptive Characteristics	Groups		*p* Value
	The Group with Spontaneous Abortions	The Group with Normal Deliveries	
Settlements Near Agricultural Lands (n/%)	49 (62.8%) *	50 (42%)	*p* < 0.004
Settlements Far from Agricultural Lands (n/%)	29 (37.2%)	69 (58%)	
Pesticide Exposure History	69 (42.9%)	31 (79.5%)	*p* < 0.000
	92 (57.1%)	8 (20.5%)	
Educational Status (n/%)	Literate 2 (2%)	Literate 4 (4%)	*p* > 0.05
	Primary school graduate 43 (43%)	Primary school graduate 34 (34%)	
	Secondary/high school graduate 52 (52%)	Secondary/high school graduate 55 (55%)	
	Undergraduate education and above 3(3%)	Undergraduate education and above 7 (7%)	
Spontaneous Abortion Before 12 weeks Spontaneous Abortion After 12 weeks	73(73%) 27(27%)		
Birth Weight	-	<2500 g- 5 (%5) 2500–3000 g- 6 (%6) 3000–3500 g- 50 (%50) >3500 g- 39 (%39)	
Total	100	100	

*: Column percentage.

**Table 3:** Distribution and comparison of OCP and PCB levels (ng/mL) in case and control groups.

OC Pesticides– PCBs	The Group with Spontaneous Abortions				The Group with Normal Deliveries				
	Mean ± SD (ng/mL)	25%	50%	75%	Mean ± SD (ng/mL)	25%	50%	75%	*p* Value
γ-HCH	51.5 ± 42.5	25.0	30.8	57.3	32.6 ± 44.7	0.0	21.8	37.3	0.799
β-HCH	7.8 ±2.9	5.2	7.7	10.6	8.6 ± 3.0	6.4	6.7	11.9	0.327
δ-HCH	31.7 ± 26.6	7.8	33.9	54.5	35.4 ± 57.9	7.3	11.1	75.7	0.754
PCB-52	20.8 ± 26.1	5.7	10.0	28.5	13.5 ± 11.9	6.4	9.7	22.6	1.0
PCB-28	10.5 ± 5.5	7.4	9.1	11.8	8.8 ± 5.0	7.0	8.9	10.1	0.39
PCB-118	18.7 ± 14.2	9.1	11.6	31.8	29.0 ± 46.1	7.2	14.2	25.4	1.0
PCB-101	11.5 ± 5.4	8.1	9.5	14.4	5.5 ± 0.7	5.5	5.0	-	0.059
PCB-153	122 ± 72.6	50	135	192	169 ± 74.2	94.7	194	232	0.291
PCB-138	11.3 ± 6.7	6.1	10.5	14.1	13.1 ± 8.2	7.0	8.6	20.4	0.590
PCB-180	11.1 ± 8.5	6.4	8.4	11.8	8.1 ± 2.9	5.9	7.4	9.3	0.252
PCB-202	7.9 ± 2.2	6.4	7.2	9.8	9.6 ± 2.8	7.0	9.9	12.0	0.123
op′-DDE	26.7 ± 32.8	9.7	16.31	29.1	27.8 ± 12.4	20.3	25.5	31.7	0.128
op′-DDT	61.6 ± 27.7	42.0	61.6	0	19.4 ± 9.7	10.9	18.8	28.5	0.064
op′-DDD	22.5 ± 15.9	11.5	17.5	31.6	34.6 ± 32.7	11.8	23.6	43.4	0.468
pp′-DDE	30.7	30.7	30.7	30.7	6.1	6.1	6.1	6.1	0.317
pp′-DDT	37.39 ± 45.7	12.8	19.4	42.5	59.5 ± 44.3	21.2	42.4	79.6	0.053
pp′-DDD	52.1 ± 57.2	14.5	26.2	78.1	66.6 ± 69.0	11.7	39.2	103.6	0.574

SD: Standard Deviation.

**Table 4:** Relationship between residual pesticides, PCBs, and groups.

Pesticides and PCBs	Groups	n	%	ERR *	*p* Value **
γ-HCH	The group with spontaneous abortions	29	67.4%	0.62	0.01
	The group with normal deliveries	14	32.6%		
PCB-52	The group with spontaneous abortions	18	69.2%	0.39	0.036
	The group with normal deliveries	8	30.8%		
PCB-101	The group with spontaneous abortions	13	86.7%	0.13	0.003
	The group with normal deliveries	2	14.3%		
PCB-138	The group with spontaneous abortions	26	63.4%	0.50	0.05
	The group with normal deliveries	15	36.6%		
Pentimanthalin	The group with spontaneous abortions	0		-	0.013
	The group with normal deliveries	6	6%		

ERR *: estimated relative risk; ** Chi-Square *p* value.

## Data Availability

The datasets are available from the corresponding author upon reasonable request.

## Supplementary Materials

The following supporting information can be downloaded at https://www.mdpi.com/article/10.3390/toxics11110884/s1, Table S1: analysis parameters for LC-MS/MS instruments of organophosphorus pesticides (OPs); Table S2: analysis parameters for gas chromatography device of organochlorine compounds (OCs).

## References

[1] Rossen L.M., Ahrens K.A., Branum A.M. (2018). Trends in Risk of Pregnancy Loss Among US Women, 1990–2011. Paediatr. Perinat. Epidemiol..

[2] La X., Wang W., Zhang M., Liang L. (2021). Definition and Multiple Factors of Recurrent Spontaneous Abortion. Adv. Exp. Med. Biol..

[3] Griebel C.P., Halvorsen J., Golemon T.B., Day A.A. (2005). Management of spontaneous abortion. Am. Fam. Physician.

[4] Mazziotta C., Pellielo G., Tognon M., Martini F., Rotondo J.C. (2021). Significantly Low Levels of IgG Antibodies Against Oncogenic Merkel Cell Polyomavirus in Sera from Females Affected by Spontaneous Abortion. Front. Microbiol..

[5] Carlsson I., Breding K., Larsson P.G. (2018). Complications related to induced abortion: A combined retrospective and longitudinal follow-up study. BMC Women’s Health.

[6] Tudi M., Daniel Ruan H., Wang L., Lyu J., Sadler R., Connell D., Chu C., Phung D.T. (2021). Agriculture Development, Pesticide Application and Its Impact on the Environment. Int. J. Environ. Res. Public Health.

[7] Altıkat A., Turan T., Torun F.E., Bingül Z. (2009). Türkiye’de pestisit kullanımı ve çevreye olan etkileri. Atatürk Üniv. Ziraat Fakültesi Derg..

[8] Zhu W., Zheng H., Liu J., Cai J., Wang G., Li Y., Shen H., Yang J., Wang X., Wu J. (2022). The correlation between chronic exposure to particulate matter and spontaneous abortion: A meta-analysis. Chemosphere.

[9] Foster W.G., Gannon A.-M., Skinner M.K. (2018). Reproductive Toxicity of Environmental Contaminants in the Female. Encyclopedia of Reproduction.

[10] Llop S., Ballester F., Vizcaino E., Murcia M., Lopez-Espinosa M.J., Rebagliato M., Vioque J., Marco A., Grimalt J.O. (2010). Concentrations and determinants of organochlorine levels among pregnant women in Eastern Spain. Sci. Total Environ..

[11] Prahl M., Odorizzi P., Gingrich D., Muhindo M., McIntyre T., Budker R., Jagannathan P., Farrington L., Nalubega M., Nankya F. (2021). Exposure to pesticides in utero impacts the fetal immune system and response to vaccination in infancy. Nat. Commun..

[12] Jaacks L.M., Diao N., Calafat A.M., Ospina M., Mazumdar M., Ibne Hasan M.O.S., Wright R., Quamruzzaman Q., Christiani D.C. (2019). Association of prenatal pesticide exposures with adverse pregnancy outcomes and stunting in rural Bangladesh. Environ. Int..

[13] Kalliora C., Mamoulakis C., Vasilopoulos E., Stamatiades G.A., Kalafati L., Barouni R., Karakousi T., Abdollahi M., Tsatsakis A. (2018). Association of pesticide exposure with human congenital abnormalities. Toxicol. Appl. Pharmacol..

[14] Pascale A., Laborde A. (2020). Impact of pesticide exposure in childhood. Rev. Environ. Health.

[15] Settimi L., Spinelli A., Lauria L., Miceli G., Pupp N., Angotzi G., Fedi A., Donati S., Miligi L., Osborn J. (2008). Spontaneous abortion and maternal work in greenhouses. Am. J. Ind. Med..

[16] Han X., Lu T., Hu Y., Duan J., Guan Y., Huang X., Zhou J., Huang R., Tang M., Sun R. (2022). A metabolomic study on the effect of prenatal exposure to Benzophenone-3 on spontaneous fetal loss in mice. Ecotoxicol. Environ. Saf..

[17] He Q.L., Zhang L., Liu S.Z. (2021). Effects of Polychlorinated Biphenyls on Animal Reproductive Systems and Epigenetic Modifications. Bull. Environ. Contam. Toxicol..

[18] Björvang R.D., Hassan J., Stefopoulou M., Gemzell-Danielsson K., Pedrelli M., Kiviranta H., Rantakokko P., Ruokojärvi P., Lindh C.H., Acharya G. (2021). Persistent organic pollutants and the size of ovarian reserve in reproductive-aged women. Environ. Int..

[19] Erdfelder E., Faul F., Buchner A. (1996). GPOWER: A general power analysis program. Behav. Res. Methods Instrum. Comput..

[20] Porta M., Pumarega J., Gasull M. (2012). Number of persistent organic pollutants detected at high concentrations in a general population. Environ. Int..

[21] Jain D., Kumar Verma R., Sharma V., Kaur A., Rai A.R., Kumari P., Nagar V., Singh Sankhla M., Parihar K. (2023). Associations between high levels pesticide and adverse reproductive outcomes in females: A comprehensive review. Mater. Today Proc..

[22] Addissie Y.A., Kruszka P., Troia A., Wong Z.C., Everson J.L., Kozel B.A., Lipinski R.J., Malecki K.M.C., Muenke M. (2020). Prenatal exposure to pesticides and risk for holoprosencephaly: A case-control study. Environ. Health.

[23] El-Baz M.A.H., Amin A.F., Mohany K.M. (2023). Exposure to pesticide components causes recurrent pregnancy loss by increasing placental oxidative stress and apoptosis: A case-control study. Sci. Rep..

[24] Aktar W., Sengupta D., Chowdhury A. (2009). Impact of pesticides use in agriculture: Their benefits and hazards. Interdiscip. Toxicol..

[25] Kapeleka J.A., Sauli E., Sadik O., Ndakidemi P.A. (2019). Biomonitoring of Acetylcholinesterase (AChE) Activity among Smallholder Horticultural Farmers Occupationally Exposed to Mixtures of Pesticides in Tanzania. J. Environ. Public Health.

[26] Pandey A., Jaiswar S.P., Ansari N.G., Deo S., Sankhwar P., Pant S., Upadhyay S. (2020). Pesticide Risk and Recurrent Pregnancy Loss in Females of Subhumid Region of India. Niger. Med. J..

[27] Arbuckle T.E., Lin Z., Mery L.S. (2001). An exploratory analysis of the effect of pesticide exposure on the risk of spontaneous abortion in an Ontario farm population. Environ. Health Perspect..

[28] Rita P., Reddy P.P., Reddy S.V. (1987). Monitoring of workers occupationally exposed to pesticides in grape gardens of andhra pradesh. Environ. Res..

[29] Piazza M.J., Urbanetz A.A. (2019). Environmental toxins and the impact of other endocrine disrupting chemicals in women’s reproductive health. JBRA Assist. Reprod..

[30] Öztekin O., Köken R., Bulut S., Alpay F. (2011). Quantification of Pesticides Levels in Mother Milk in the City of Afyonkarahisar and Its Epidemiological Influence. Turk. Klin. J. Pediatr..

[31] Korrick S.A., Chen C., Damokosh A.I., Ni J., Liu X., Cho S.-I., Altshul L., Ryan L., Xu X. (2001). Association of DDT with spontaneous abortion: A case-control study. Ann. Epidemiol..

[32] Longnecker M.P., Klebanoff M.A., Dunson D.B., Guo X., Chen Z., Zhou H., Brock J.W. (2005). Maternal serum level of the DDT metabolite DDE in relation to fetal loss in previous pregnancies. Environ. Res..

[33] Ruan F., Liu C., Hu W., Ruan J., Ding X., Zhang L., Yang C., Zuo Z., He C., Huang J. (2022). Early life PCB138 exposure induces kidney injury secondary to hyperuricemia in male mice. Environ. Pollut..

[34] Montano L., Pironti C., Pinto G., Ricciardi M., Buono A., Brogna C., Venier M., Piscopo M., Amoresano A., Motta O. (2022). Polychlorinated Biphenyls (PCBs) in the Environment: Occupational and Exposure Events, Effects on Human Health and Fertility. Toxics.

[35] Lefebvre T., Fréour T., Duval G., Ploteau S., Marchand P., Le Bizec B., Antignac J.-P., Cano-Sancho G. (2022). Associations between internal concentrations of fluorinated and organochlorinated chemicals in women and in vitro fertilization outcomes: A multi-pollutant study. Environ. Pollut..

[36] Meng Y., Zhong K., Chen S., Huang Y., Wei Y., Wu J., Liu J., Xu Z., Guo J., Liu F. (2021). Cardiac toxicity assessment of pendimethalin in zebrafish embryos. Ecotoxicol. Environ. Saf..

[37] El-Sharkawy N.I., Reda R.M., El-Araby I.E. (2011). Assessment of Stomp^®^(Pendimethalin) toxicity on Oreochromis niloticus. J. Am. Sci..

[38] Giglio A., Vommaro M.L. (2022). Dinitroaniline herbicides: A comprehensive review of toxicity and side effects on animal non-target organisms. Environ. Sci. Pollut. Res. Int..

[39] Hou L., Lee W.J., Rusiecki J., Hoppin J.A., Blair A., Bonner M.R., Lubin J.H., Samanic C., Sandler D.P., Dosemeci M. (2006). Pendimethalin exposure and cancer incidence among pesticide applicators. Epidemiology.

[40] Li Y., Macdonald R. (2005). Sources and pathways of selected organochlorine pesticides to the Arctic and the effect of pathway divergence on HCH trends in biota: A review. Sci. Total Environ..

[41] Agrahari A., Singh A., Srivastava A., Jha R.R., Patel D.K., Yadav S., Srivastava V., Parmar D. (2019). Overexpression of cerebral cytochrome P450s in prenatally exposed offspring modify the toxicity of lindane in rechallenged offspring. Toxicol. Appl. Pharmacol..

[42] Ben Mukiibi S., Nyanzi S.A., Kwetegyeka J., Olisah C., Taiwo A.M., Mubiru E., Tebandeke E., Matovu H., Odongo S., Abayi J.J.M. (2021). Organochlorine pesticide residues in Uganda’s honey as a bioindicator of environmental contamination and reproductive health implications to consumers. Ecotoxicol. Environ. Saf..

[43] Pathak R., Mustafa M.D., Ahmed R.S., Tripathi A.K., Guleria K., Banerjee B.D. (2010). Association between recurrent miscarriages and organochlorine pesticide levels. Clin. Biochem..

[44] Arbuckle T.E., Savitz D.A., Mery L.S., Curtis K.M. (1999). Exposure to Phenoxy Herbicides and the Risk of Spontaneous Abortion. Epidemiology.

[45] Zhang X., Fan J., Chen Y., Wang J., Song Z., Zhao J., Li Z., Wu X., Hu Y. (2021). Cytogenetic Analysis of the Products of Conception after Spontaneous Abortion in the First Trimester. Cytogenet. Genome Res..

[46] Öztekin L. (2005). Şeftali ve Şeftali Sularında Bazı Organik Fosforlu ve Bromlu Pestisit Kalıntılarının Saptanması. Ph.D. Dissertation.

[47] Nguyen Dang Giang C., Le D.B.C., Nguyen V.H., Hoang T.L., Tran T.V.T., Huynh T.P.L., Nguyen T.Q.T. (2022). Assessment of pesticide use and pesticide residues in vegetables from two provinces in Central Vietnam. PLoS ONE.

[48] Çatak H., Polat B., Tiryaki O. (2020). Farklı yıkama uygulamaları ile kapya biberlerde pirimiphos-methyl kalıntısının giderilmesi. Anadolu Tarım Bilim. Derg..

[49] Yang S.-J., Mun S., Kim H.J., Han S.J., Kim D.W., Cho B.-S., Kim A.G., Park D.W. (2022). Effectiveness of Different Washing Strategies on Pesticide Residue Removal: The First Comparative Study on Leafy Vegetables. Foods.

